# Population Genetic Structure, Historical Effective Population Size, and Dairy Trait Selection Signatures in Chinese Red Steppe and Holstein Cattle

**DOI:** 10.3390/ani15172516

**Published:** 2025-08-27

**Authors:** Peng Niu, Xiaopeng Li, Xueyan Wang, Huimin Qu, Hong Chen, Fei Huang, Kai Hu, Di Fang, Qinghua Gao

**Affiliations:** 1 College of Life Science and Technology, Tarim University, Alar 843300, China; npn0805@126.com (P.N.); chenhong090588@163.com (H.C.); huangf21@126.com (F.H.); 2 College of Animal Science and Technology, Tarim University, Alar 843300, China; miexiaochi@163.com (X.L.); wangxueyan1016@163.com (X.W.); quhuimin1128@126.com (H.Q.); hk20012022@163.com (K.H.); difangtj@163.com (D.F.); 3 Key Laboratory of Livestock and Grass Resources Utilization Around Tarim, Ministry of Agriculture and Rural Areas, Alar 843300, China

**Keywords:** Chinese red steppe cattle, Holstein cattle, population structure, effective population size, SMC++, FST, XP-CLR, butanoate metabolism, *ACSM* genes

## Abstract

Background: Chinese Red Steppe cattle (CRS) combine indigenous environmental resilience with moderate dairy performance, whereas Holstein cattle (HOL), despite their high milk yield, suffer reduced genetic diversity and compromised adaptation. A comparative analysis of their population genetic architecture and selection signatures can reveal valuable targets for CRS dairy improvement. Methods: We genotyped 61 CRS and 392 HOL individuals using the Illumina GGP Bovine 100K SNP array and performed stringent quality control. Population structure was assessed via principal component analysis, neighbor-joining trees, and sparse nonnegative matrix factorization. Historical effective population size (Ne) and divergence time were inferred with SMC++. Genome-wide selection scans combined Fixation Index (FST) and Cross-Population Composite Likelihood Ratio test (XP-CLR); overlapping high-confidence regions were annotated and subjected to GO and KEGG enrichment analyses. Results: CRS and HOL were clearly separated along PC1 (explaining 57.48% of variance), with CRS exhibiting high internal homogeneity and weak substructure, versus greater diversity and complex substructure in HOL. SMC++ indicated a split approximately 3500 years ago (700 generations) and a pronounced recent decline in Ne for both breeds. Joint selection mapping identified 767 candidate genes; notably, the *ACSM1/2B/3/4* cluster on chromosome 25—key to butanoate metabolism—showed the strongest signal. Enrichment analyses highlighted roles for proteasome function, endoplasmic reticulum stress response, ion homeostasis, and RNA processing in regulating milk fat synthesis and protein secretion. Conclusion: This study delineates the genetic divergence and demographic history of CRS and HOL, and pinpoints core genes and pathways—particularly those governing butanoate metabolism and protein quality control—underlying dairy traits. These findings furnish molecular markers and theoretical guidance for precision breeding and sustainable utilization of Chinese Red Steppe cattle.

## Simple Summary

Chinese Red Steppe cattle (CRS) are well adapted to local environments but have moderate milk yields, while Chinese Holsteins (HOL) deliver high milk production at the cost of genetic diversity and adaptability. By comparing 61 CRS and 392 HOL using genome-wide SNP data, we characterized their genetic structure, traced their demographic histories, and identified regions under selection. We found that the ancestors of CRS and HOL diverged about 3500 years ago and that both breeds have experienced recent declines in population size. Importantly, we pinpointed 767 candidate genes, with a cluster of ACSM family genes on chromosome 25—central to butanoate (butyrate) metabolism—showing the strongest selection signal. We also uncovered selection on pathways involved in protein folding, ion balance, and RNA processing, which are all crucial for milk synthesis. These findings provide potential genetic targets for improving milk production in CRS, which could be further validated through functional studies and incorporated into genomic selection programs, thereby enhancing production traits while preserving the breed’s valuable adaptive characteristics.

## 1. Introduction

Dairy cattle hold significant economic value in global livestock industries, with their dairy products directly impacting human nutrition and the development of the food industry [1,2,3]. However, while Holstein cattle are known for their high milk yield, prolonged intensive breeding has led to a decline in genetic diversity and limited environmental adaptability. They exhibit instability under conditions of high temperature, cold, and roughage feeding, which restricts their sustainable development [4,5,6]. In contrast, the Chinese Red Steppe (CRS), a composite breed formed through years of local selection, possesses both decent milk production and excellent environmental adaptability. Widely distributed in cold, arid regions with abundant grassland resources, CRS holds potential for developing local, adaptive dairy resources [7].

Studies on the genetic diversity and population structure of CRS have shown high internal diversity and low inbreeding levels, providing essential information for breeding [8,9]. In contrast, multiple studies on Holstein cattle have revealed a decline in effective population size (Ne) under the pressure of high-yield breeding, which affects resilience and health traits, indicating that both historical population dynamics and modern breeding exert a profound impact on genetic resources [5,10]. Methods like SMC++ that reconstruct Ne history and estimate divergence times based on whole-genome data have revealed events related to domestication, breeding, and environmental adaptation in various cattle breeds [11,12,13]. However, there is a notable gap in comparative studies of the system-level Ne between CRS and Holstein. Furthermore, whole-genome selection signal detection methods have successfully identified genes related to milk yield, milk fat synthesis, mammary gland development, and energy metabolism in dairy traits research [14].

FST (Fixation Index) is a widely used method to assess genetic differentiation between populations by comparing allele frequencies across loci [15]. This approach is particularly useful for identifying genomic regions exhibiting significant differentiation, which may reflect selective pressures. However, FST alone can inevitably produce false positives, especially when there is incomplete differentiation between populations or when the population size is small [16]. XP-CLR (Cross-Population Composite Likelihood Ratio), on the other hand, is particularly effective in detecting selective sweeps by comparing allele frequencies between two populations. This method is based on the Site Frequency Spectrum (SFS), which provides a detailed picture of genetic variation within populations [17]. XP-CLR can more accurately identify regions that have experienced recent positive selection, which often leads to large allele frequency differences between populations [18]. By focusing on the SFS, XP-CLR can effectively detect selection signatures in regions that show stark frequency differences between populations, helping to reduce the risk of false positives that may arise from FST when the genetic differentiation is subtle.

This study aims to use high-density SNP chips and whole-genome data to conduct population structure analysis, SMC++ historical Ne reconstruction, and divergence time estimation for CRS (*n* = 61) and Holstein (*n* = 392). Additionally, Fst and XP-CLR methods will be employed to detect selection signals related to dairy traits. Functional annotation and enrichment analysis will be used to identify potential dairy improvement resources in CRS. The goal is to provide theoretical support for the genetic improvement of Chinese Red Steppe and the sustainable use of local resources, as well as scientific evidence for developing cross-breed complementary breeding strategies.

## 2. Materials and Methods

### 2.1. Samples and Sequencing Data

This study includes a total of 453 cattle, including 61 Chinese Red Steppe (CRS) cattle (Figshare: https://doi.org/10.6084/m9.figshare.21586182.v1, accessed on 19 June 2025) and 392 Holstein cattle (genome variation map (GVM) accession number: GVM000516). Genotyping of CRS and Holstein individuals was performed using SNP chips (Illumina GGP Bovine 100K genotyping data). All samples were aligned and annotated using the ARS-UCD1.2 reference bovine genome.

### 2.2. Data Quality Control

The raw genotype data were merged and quality-controlled using PLINK [19] (v1.90). First, SNPs with a missing rate greater than 5% were filtered using the --geno 0.05 command to remove markers likely caused by genotyping failure or low quality. Similarly, individuals with a missing rate greater than 5% were filtered using the --mind 0.05 command to exclude samples with poor quality or potential experimental issues. For low-frequency variants, SNPs with a minor allele frequency (MAF) below 0.01 were removed using the --maf 0.01 command to reduce statistical instability and potential genotyping errors caused by rare variants. Finally, Hardy–Weinberg equilibrium tests were applied to filter out significantly deviating SNPs (--hwe 1 × 10^−6^), excluding markers potentially affected by genotyping errors or strong selective pressure.

### 2.3. Population Genetic Structure Analysis

To investigate the genetic structure, we conducted Neighbor-Joining (NJ) phylogenetic analysis, principal component analysis (PCA), and ancestral proportion estimation using sparse non-negative matrix factorization (sNMF) and least-squares optimization. PCA was performed on the quality-controlled SNP data using PLINK software. The NJ tree was constructed based on the p-distance matrix, which was calculated using VCF2Dis (v1.54) (https://github.com/BGI-shenzhen/VCF2Dis accessed on 19 June 2025), and the tree was visualized using the iTOL tool (version 7.2.1: https://itol.embl.de/upload.cgi) [20].

For admixture analysis, linkage disequilibrium (LD) pruning was carried out in PLINK with a sliding window of 50 SNPs and a step size of 5 SNPs. SNPs with an r^2^ greater than 0.2 within each window (-indep-pairwise 50 5 0.2) were removed, and the SNP with the highest minor allele frequency (MAF) was retained within each LD block. After pruning, we tested K values ranging from 2 to 8. The most likely number of ancestral populations was determined by minimizing the cross-validation error, and the results were visualized using the pong tool (https://github.com/ramachandran-lab/pong accessed on 19 June 2025).

Effective population size (Ne) analysis was conducted using SMC++ (v1.15.2) [21]. Prior to analysis, VCF files were converted into the SMC++-specific format, with a mutation rate (μ) of 1.2 × 10^−8^ and a generation interval (g) of 5 years. Model smoothing was controlled using the --knots 8 option, and the regularization penalty was adjusted based on the fit. Single-population modeling was performed for both CRS and Holstein to reconstruct their historical Ne trajectories. The smc++ split command was used to analyze the divergence history of the two populations and estimate their divergence time.

### 2.4. Identification of Selective Sweeps

Based on VCFtools (version 0.1.16) [22], Weir and Cockerham’s FST values were calculated for both single-SNP and sliding window analyses, with a window size of 1 Mb and a step size of 5 kb. The top 5% of windows and single SNPs with the highest FST values were selected as candidate regions under selection, and their chromosomal locations and significant segments were recorded.

The XP-CLR (Cross-Population Composite Likelihood Ratio) method [18] was used to detect selection sweeps by modeling the multi-locus allele frequency differences between the two populations and constructing a composite likelihood ratio statistic. This method has a high detection power for complex population structures and can be applied to unphased data.

In this study, XP-CLR (v1.1.2) software was used to implement the algorithm proposed by [23] to compare selection signals between the Chinese Red Steppe (CRS) and Holstein (HOL) populations. The window size was set to 1 Mb with a step size of 5 kb; the maximum number of SNPs per window (--maxsnps) was set to 1000 to balance signal detection sensitivity and computational efficiency; and the minimum number of SNPs per window (--minsnps) was set to 10 to avoid statistical instability due to sparse windows. The XP-CLR scores for each chromosome were recorded in score files, detailing the position and corresponding score of each sliding window. After merging the results from all chromosomes, the top 5% of high-signal windows were selected as candidate regions. Finally, these candidate regions were overlapped with the FST analysis results, and intersecting regions were identified to enhance signal reliability.

### 2.5. Candidate Region Annotation and Functional Enrichment Analysis

After converting the candidate selection regions into BED format, the Bedtools intersect command was used to perform an intersection operation with the gene annotation file (GFF3) of the ARS-UCD1.2 reference genome, identifying the candidate genes within these regions. The overlapping genes from both FST and XP-CLR results were combined, and duplicates were removed to form a final list of candidate genes. A brief functional description of each gene was then extracted.

Further, GO and KEGG functional enrichment analyses of the candidate genes were conducted using the clusterProfiler package in R [24]. This analysis aimed to identify the biological processes, molecular functions, and pathways that the candidate genes are involved in, helping to uncover potential functional roles related to dairy traits such as milk yield, composition, and mammary gland development.

## 3. Results and Analysis

Quality control was performed on the merged dataset of 453 samples and 80,853 SNPs using PLINK, with the following thresholds: missing data filtering (SNP missing rate > 5%: --geno 0.05; sample missing rate > 5%: --mind 0.05), minor allele frequency (MAF) filtering (MAF < 0.01: --maf 0.01), and Hardy–Weinberg equilibrium (HWE) testing (*p* < 1 × 10^−6^: --hwe 1 × 10^−6^). The results showed that no samples were excluded due to excessive missing data. A total of 813 variants were removed due to missing rate filtering, and 21 variants were removed based on the HWE test. No additional variants were excluded due to the MAF filtering. After quality control, 453 samples and 80,019 high-quality SNPs were retained, with an overall genotyping rate of 0.9966. This quality control process ensured that the subsequent population structure and selection signal analyses were based on reliable genotypic data.

### 3.1. Population Structure

**Figure 1:**
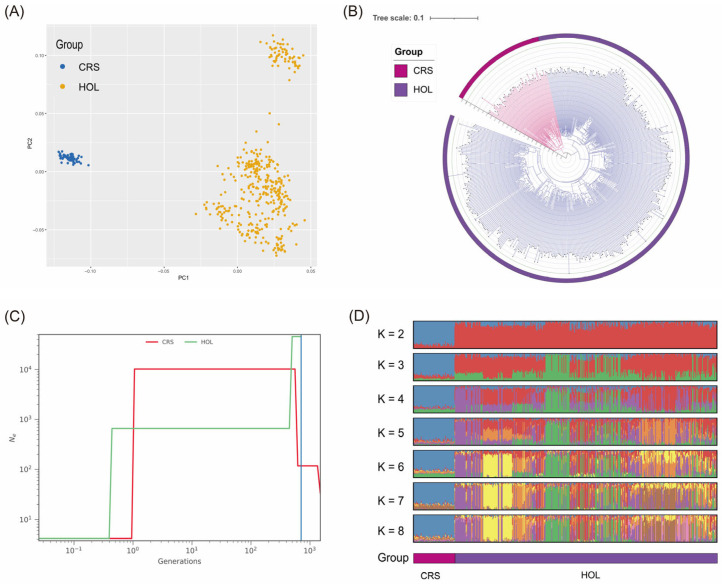
Population Structure Analysis Results. (**A**) PCA Scatter Plot. (**B**) NJ Neighbor-Joining Tree. (**C**) Historical Effective Population Size (Ne) and Divergence Time Reconstruction. (**D**) sNMF Ancestry Proportion Bar Plot: The x-axis represents all individuals, ordered by population (CRS and HOL), and the y-axis represents the proportion of each ancestry component.

PCA showed significant separation between CRS and HOL along PC1, with PC1 explaining 57.48% of the total variance and PC2 explaining 42.52% of the variance, which helped to reveal the internal substructure (Figure 1A). CRS samples clustered in the negative region of PC1 with a narrow variance range, indicating higher genetic homogeneity within the population. In contrast, HOL samples were distributed in the positive region of PC1 and were relatively dispersed, suggesting greater genetic diversity within the population. The NJ tree also clearly differentiated the two populations, with branch lengths primarily concentrated between 0.17 and 0.19, indicating a similar degree of genetic drift between CRS and HOL. Some individual branches exceeded 0.2, suggesting these samples may have stronger differentiation signals or unique genetic backgrounds, requiring further validation based on sample sources and data quality (Figure 1B).

The sNMF ancestry component analysis at K = 2 clearly separated CRS and HOL, with CRS predominantly composed of a single ancestral component and HOL predominantly composed of another, with minimal admixture (Figure 1C). As K increased to 3–5, multiple ancestral components appeared within HOL, indicating the presence of subgroups or different breeding lines, with varying degrees of admixture between individuals. In contrast, CRS remained largely homogeneous with a dominant single component at higher K values, and only a small amount of minor components appeared at K ≥ 6, suggesting limited substructure. Cross-validation error in sNMF showed a clear inflection point at K = 2 or 3, indicating that the primary number of populations is low, but the substructure within HOL could be further refined at higher K values (Figure 1D).

Historical Ne reconstruction and divergence time estimation indicated that ancient CRS and HOL shared a high Ne before diverging. CRS experienced a slight expansion before and after the divergence but remained stable, with a notable decline in recent times. HOL also underwent an expansion phase post-divergence, but with different timing and extent compared to CRS, with a more significant recent Ne decline. Based on SMC++ split analysis, the divergence between CRS and HOL occurred approximately 700 generations ago (around 3500 years ago), consistent with the domestication and migration history of cattle (Figure 1C). The recent Ne decline may reflect the increased intensity of modern breeding and population size constraints, highlighting the need to focus on genetic diversity conservation and breeding planning (Supplementary Table S1).

### 3.2. Selection Signal Detection

**Figure 2:**
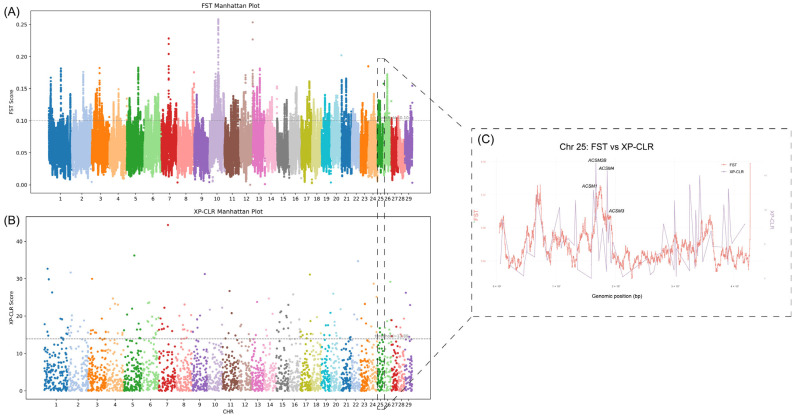
(**A**) Whole-genome FST Manhattan Plot: The x-axis represents the positions of sliding windows (1 Mb window, 5 kb step) ordered by chromosome, with alternating colors distinguishing different chromosomes. The y-axis shows the Weir–Cockerham FST values. The dashed line marks the threshold for the top 5% of FST windows. (**B**) Whole-genome XP-CLR Manhattan Plot: The x-axis shows the chromosome positions, with different colors used to distinguish the chromosomes. The y-axis represents the XP-CLR scores. The dashed line marks the threshold for the top 5% of XP-CLR windows. Certain regions show consistent high scores in both FST and XP-CLR analyses, supporting the presence of selection signals. (**C**) Local Enlargement of the Significant Region on Chromosome 25: The x-axis represents the genomic positions on chromosome 25, with the left y-axis showing the sliding window FST values (orange) and the right y-axis showing the XP-CLR scores (purple).

The results of the whole-genome Weir–Cockerham FST sliding window analysis (Figure 2A) revealed significant peaks of differentiation on several chromosomes. FST values above the threshold were observed in 5011 genes (Supplementary Table S2). The XP-CLR scan (Figure 2B) identified 2299 genes (Supplementary Table S3), with some overlap with the FST peaks on certain chromosomes. Based on the predefined threshold (top 5% of window scores), candidate regions from both FST and XP-CLR were extracted and subsequently analyzed for overlap, resulting in 767 genes.

On chromosome 25, both FST and XP-CLR showed synchronized peaks (Figure 2C). A zoomed-in view (Figure 2C) reveals that the region in the FST scan scored significantly higher than the baseline, and the XP-CLR score also reached a high level across the entire genome, indicating strong evidence of selective differentiation. Annotation results indicated that the peak region encompasses genes such as *ACSM2B*, *ACSM1*, *ACSM4*, and *ACSM3*, which are involved in the activation and metabolism of medium-chain fatty acids. These genes may be related to milk fat synthesis or energy utilization. This region shows clear differentiation between CRS and HOL populations, suggesting differences in selection pressures related to dairy traits or metabolic characteristics.

### 3.3. Enrichment Analysis of Milk Production-Related Pathways and Regulatory Genes

**Figure 3:**
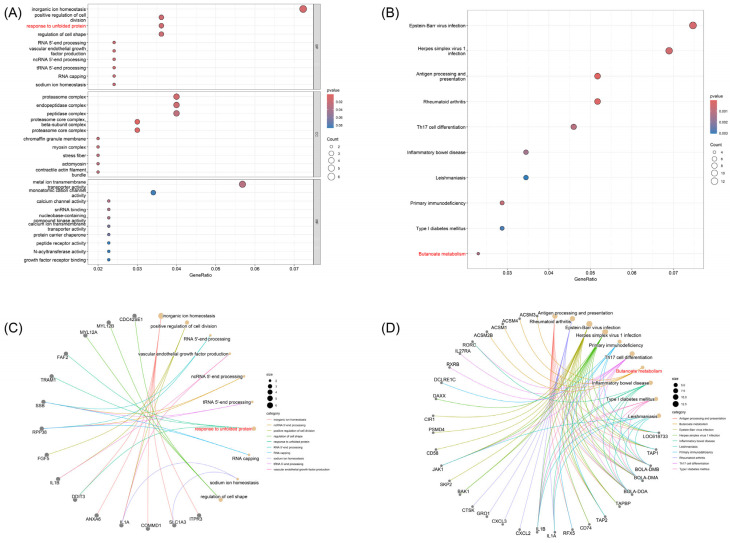
Overview of Candidate Gene GO and KEGG Enrichment Analysis. (**A**) GO Enrichment Analysis Bubble Plot: The three categories of GO terms (Biological Process, Cellular Component, and Molecular Function) are shown. The x-axis represents the-log10 (*p*-value), and the y-axis lists the significant GO terms. The color of the bubbles ranges from red to blue, indicating *p*-values from large to small (dark blue represents more significant terms). The size of each bubble reflects the number of candidate genes enriched in that GO term. (**B**) KEGG Pathway Enrichment Bubble Plot: The x-axis is also-log10 (*p*-value), and the y-axis represents significant pathways. The size of the bubbles indicates the number of candidate genes involved, and the color represents the significance of the enrichment. (**C**) GO Pathway–Gene Network Diagram: Yellow solid circular nodes represent the GO pathways, and gray solid circular nodes represent the genes. The connecting lines show the participation of genes in the corresponding GO terms. The term “response to unfolded protein” is highlighted in red to emphasize its key role in the candidate gene set. (**D**) KEGG Pathway–Gene Network Diagram: Yellow solid circular nodes represent KEGG pathways, and gray solid circular nodes represent genes. Connecting lines indicate participation relationships. The “Butanoate metabolism” pathway is highlighted in red, with the ACSM family gene region prominently displayed, underscoring its potential functional role in milk fat metabolism.

The GO analysis revealed significant enrichment in several functional categories relevant to milk production. In the cellular component category, the terms “proteasome core complex” (GO:0000502) and “proteasome core complex, beta-subunit complex” (GO:0019774) were enriched, corresponding to the genes *PSMB8*, *PSMB9*, *PSMB4*, and *PSMD4*. In the biological process category, “response to unfolded protein” (GO:0006986) was enriched, encompassing the genes *TRAM1, FAF2,* and *DDIT3*. During peak lactation, the mammary gland must synthesize and secrete large amounts of casein and whey proteins, which significantly increases the load on the endoplasmic reticulum. The optimization of the proteasome and the folding monitoring system plays a crucial role in maintaining the secretion pathway and reducing cellular stress (Figure 3A,C).

In the biological process category, “inorganic ion homeostasis” (GO:0098771) and “sodium ion homeostasis” (GO:0055078) were enriched, with genes such as *ITPR3, SLC1A3*, *COMMD1*, *IL1A*, *ANXA6*, and *DDIT3* involved. Regulation of ion gradients, such as sodium and calcium, is vital for osmotic pressure, milk secretion, and electrolyte composition in mammary epithelial cells. Transport channels and signaling regulators such as *SLC1A3* and *ITPR3* may affect the sodium-calcium balance within mammary cells, thereby influencing lactation efficiency (Figure 3C).

Several GO terms related to RNA 5′-end processing (e.g., GO:0000966, GO:0036260) were enriched in genes *RPP38* and *SSB*. The rapid production and stability of high levels of milk protein mRNA are critical for maintaining continuous high milk yield. RNA precursor splicing and processing factors enhance post-transcriptional regulation and translation initiation efficiency, significantly boos24ting the protein synthesis capacity of mammary glands (Supplementary Table S4).

KEGG pathway enrichment analysis identified the “Butyrate metabolism” pathway (KEGG:bta00650) as significantly enriched with genes *ACSM2B*, *ACSM1*, *ACSM4*, and *ACSM3*. *(*Figure 3D) These medium-chain acyl-CoA synthetases are responsible for activating butyrate produced in the rumen to butyryl-CoA, providing fatty acid synthesis substrates or participating in energy metabolism in mammary cells. Butyrate not only supplies direct material for milk fat synthesis but also upregulates lipid synthesis enzyme expression through GPR receptor-mediated signaling pathways. (Figure 3D). Thus, the differentiation of ACSM family genes may determine the variation in milk fat content between different populations (Supplementary Table S5).

## 4. Discussion

This study reveals the differences in genetic structure, historical evolution, and selection pressures between Chinese Red Steppe (CRS) and Holstein (HOL) cattle through multi-level population genetic analysis, providing new insights into the improvement of dairy traits in these two populations. First, PCA and NJ tree analyses consistently show significant genetic differentiation between CRS and HOL at the whole-genome level. CRS samples cluster in the negative region of PC1 with limited internal variation, reflecting local breeding practices and geographical isolation tendencies (Figure 1A). In contrast, HOL exhibits higher internal diversity and a complex substructure, likely due to multi-source breeding and global gene flow [25,26,27,28]. The sNMF ancestry component analysis further demonstrates that the CRS population has a single ancestral component with weak substructure, while HOL exhibits multiple ancestral components when K > 2, indicating the presence of different breeding lines or historical admixture events within HOL [25]. These findings align closely with the breeding histories of the two populations: CRS, as a local breed, has maintained relative homogeneity under extensive management over time, while HOL has accumulated more genetic variation through international crossbreeding and modern selective breeding processes [29,30,31].

The historical Ne variation and divergence time reconstruction using SMC++ revealed that the two populations began to diverge approximately 3500 years ago (700 generations), during which Ne underwent a brief expansion before stabilizing. In recent times, Ne has significantly decreased, reflecting the profound impact of human domestication and breeding interventions on population size [12,32,33,34]. Against the backdrop of increasing lactation demands and breeding intensity, the decline in Ne serves as a warning that, while pursuing high yields, attention must also be given to the conservation of genetic diversity and the adoption of rational mating strategies, in order to mitigate the adverse effects of inbreeding on health and production performance [35].

Selection signal analysis, combining FST and XP-CLR methods, identified 767 candidate genes across the entire genome, with the region on chromosome 25 containing *ACSM2B*, *ACSM1*, *ACSM4*, and *ACSM3* genes showing the most significant differentiation and selection signals. This finding is closely related to the key role of the ACSM gene family in butyrate metabolism, particularly in converting volatile fatty acids (VFA) to acyl-CoA [36,37]. After butyrate produced during rumen fermentation is converted to butyryl-CoA by the ACSM enzyme system, it can provide substrates for lipid synthesis in mammary cells or influence energy balance, thereby regulating milk fat content and lactation efficiency [38,39]. CRS, adapted to cold, arid, and rough feeding environments, faces greater selection pressure on VFA utilization efficiency, whereas HOL, selected for high milk yield, places higher demands on the rate of milk fat synthesis. This ecological and production goal difference may drive divergent selection pressures on the ACSM gene family in the two populations [40].

The enrichment analysis results indicate that two major pathways, protein folding and butyrate metabolism, play key roles in the differentiation of dairy traits between the CRS and HOL populations.

First, GO enrichment revealed significant enrichment of genes related to the terms “response to unfolded protein” (GO:0006986) and “proteasome core complex” (GO:0000502, GO:0019774), such as *TRAM1*, *FAF2*, *DDIT3*, *PSMB4*, *PSMB8*, *PSMB9*, and *PSMD4.* This suggests that during peak lactation, the mammary gland enhances the endoplasmic reticulum’s folding monitoring and proteasomal degradation mechanisms to maintain the large-scale synthesis and secretion of casein and whey proteins. This helps reduce cellular stress induced by unfolded proteins and improves milk protein yield [37,38,39].

Secondly, KEGG enrichment analysis identified the “Butyrate metabolism” (bta00650) pathway as significantly enriched with *ACSM1*, *ACSM2B*, *ACSM3*, and *ACSM4.* These enzymes activate butyrate, produced during rumen fermentation, to butyryl-CoA, which provides substrates for milk fat synthesis in mammary cells. Butyrate also regulates lipid metabolism networks such as *SREBP1* and PPARγ through GPR receptors, thereby enhancing milk fat synthesis efficiency [39,40].

In summary, these two functional modules—protein quality control and butyrate metabolism—not only provide the molecular foundation for efficient protein and lipid synthesis in lactating cells but also highlight the ACSM gene family and the proteasomal system as potential molecular markers. Future studies could verify their roles in improving milk yield and milk composition quality through expression profiling, functional knockout, or candidate gene association analysis. These findings offer new targets for precision breeding in both the Chinese Red Steppe and Holstein cattle populations.

## 5. Conclusions

This study systematically analyzed the population genetic structure, historical effective population size (Ne) reconstruction, and whole-genome selection signals of the Chinese Red Steppe (CRS) and Holstein (HOL) cattle based on Illumina GGP Bovine 100 K genotyping data. After quality control, 453 samples and 80,019 high-quality SNPs were retained, ensuring the reliability of downstream analyses. The PCA, NJ tree, and sNMF results all demonstrated significant genetic differentiation between the two populations. CRS exhibited high internal homogeneity and weak substructure, while HOL displayed greater diversity and complex subgroups. The Ne history reconstructed using SMC++ indicated that the two populations diverged approximately 3500 years ago, followed by a decline in recent Ne, reflecting the dual impact of human domestication and modern breeding on genetic resources. FST and XP-CLR joint screening identified 767 candidate genes, with the region containing ACSM family genes (*ACSM2B*/*ACSM1*/*ACSM4*/*ACSM3*) on chromosome 25 exhibiting the strongest selection signals, suggesting their potential role in medium-chain fatty acid activation and milk fat synthesis. The results not only enhance the understanding of the genetic diversity and selection history of CRS and HOL but also provide a theoretical basis for precision breeding based on metabolic pathways and local resource conservation. Future studies, combining phenotype associations and functional validation, could further explore the specific contributions of key genes to lactation performance, guiding the improvement of dairy traits and the maintenance of genetic diversity in the Chinese Red Steppe.

## Data Availability

The genotype data of Chinese Red Steppe (CRS) cattle used in this study are publicly available in the Figshare repository (https://doi.org/10.6084/m9.figshare.21586182.v1, accessed on 19 June 2025). The genotype data of Holstein (HOL) cattle were obtained from the Genome Variation Map (GVM) under accession number GVM000516.

## Supplementary Materials

The following supporting information can be downloaded at: https://www.mdpi.com/article/10.3390/ani15172516/s1, Supplementary Table S1: Results of the NE segmentation model; Supplementary Table S2: Annotation results of SNPs with the top 5% threshold in the fst category; Supplementary Table S3: Annotation results of SNPs with the top 5% threshold in the xpclr category; Supplementary Table S4: GO Enrichment Results; Supplementary Table S5: KEGG Enrichment Results.
